# Localization of near-infrared labeled antibodies to the central nervous system in experimental autoimmune encephalomyelitis

**DOI:** 10.1371/journal.pone.0212357

**Published:** 2019-02-15

**Authors:** Sangmin Lee, Hannah E. Salapa, Michael C. Levin

**Affiliations:** 1 Department of Neurology, University of Tennessee Health Science Center, Memphis, Tennessee, United States of America; 2 Office of the Saskatchewan Multiple Sclerosis Clinical Research Chair, University of Saskatchewan, Saskatoon, Saskatchewan, Canada; 3 Department of Anatomy and Cell Biology, University of Saskatchewan, Saskatoon, Saskatchewan, Canada; 4 Department of Medicine, Neurology Division, University of Saskatchewan, Saskatoon, Saskatchewan, Canada; 5 Research Service, Veterans Affairs Medical Center, Memphis, Tennessee, United States of America; University Medical Center of the Johannes Gutenberg University of Mainz, GERMANY

## Abstract

Antibodies, including antibodies to the RNA binding protein heterogeneous nuclear ribonucleoprotein A1, have been shown to contribute to the pathogenesis of multiple sclerosis, thus it is important to assess their biological activity using animal models of disease. Near-infrared optical imaging of fluorescently labeled antibodies and matrix metalloproteinase activity were measured and quantified in an animal model of multiple sclerosis, experimental autoimmune encephalomyelitis. We successfully labeled, imaged and quantified the fluorescence signal of antibodies that localized to the central nervous system of mice with experimental autoimmune encephalomyelitis. Fluorescently labeled anti-heterogeneous nuclear ribonucleoprotein A1 antibodies persisted in the central nervous system of mice with experimental autoimmune encephalomyelitis, colocalized with matrix metalloproteinase activity, correlated with clinical disease and shifted rostrally within the spinal cord, consistent with experimental autoimmune encephalomyelitis being an ascending paralysis. The fluorescent antibody signal also colocalized with matrix metalloproteinase activity in brain. Previous imaging studies in experimental autoimmune encephalomyelitis analyzed inflammatory markers such as cellular immune responses, dendritic cell activity, blood brain barrier integrity and myelination, but none assessed fluorescently labeled antibodies within the central nervous system. This data suggests a strong association between autoantibody localization and disease. This system can be used to detect other antibodies that might contribute to the pathogenesis of autoimmune diseases of the central nervous system including multiple sclerosis.

## Introduction

Multiple Sclerosis (MS) is the most common autoimmune disease of the central nervous system (CNS) in humans, affecting approximately three million people world-wide [1]. The pathogenesis of MS is complex, but data support the contribution of pathogenic T-cells, B-cells, macrophages and antibodies to demyelination and neurodegeneration (neuronal and axonal damage) in the CNS [1]. In early forms of MS, Th1 and Th17 CD4^+^ lymphocyte responses predominate and T-cells and B-cells correlate with demyelination and neuronal injury [2, 3]. With progression, the inflammatory response is diffuse (involving CNS parenchyma and meninges) and IgG-positive plasma cells predominate [2], implicating the humoral immune response in the pathogenesis of progressive MS. In support of this hypothesis are studies showing an association between antibody responses to non-myelin targets including neurofilament, neurofascin, and KIR4 [4, 5], in addition to our own data demonstrating a relationship between heterogeneous nuclear ribonucleoprotein A1 (hnRNP A1) antibodies and neurodegeneration [1, 6–8].

Experimental autoimmune encephalitis (EAE) is an animal model of MS, which is an important tool used to study the contribution of inflammation (including B-cells, antibodies, macrophages and Th1/Th17 T-cell responses) to CNS demyelination and neurodegeneration [6, 9]. In addition to immunologic and anatomic studies of EAE, a number of studies have used near infrared (nearIR) fluorescent (NIRF) optical imaging of inflammatory targets to examine the role of inflammation in the CNS. These studies include markers of T-cells [10], vascular permeability [11], blood brain barrier integrity [11, 12], matrix metalloproteinases (MMPs) [11, 12], leukocyte migration into the CNS [11, 12], dendritic cells [13], neurogenesis [14], myelination [15], and astrocyte activation [16]. Considering data supporting the contribution of immunoglobulins in the pathogenesis of MS as well as their removal improving outcomes in some MS patients [17], models that examine the role of antibodies in autoimmune disease of the CNS are important, yet none of these studies analyzed fluorescently labeled antibodies. In this study, we used a novel *ex vivo* fluorescent imaging approach to examine the macroscopic localization of anti-hnRNP A1 antibodies in EAE.

## Materials and methods

### Fluorescent agents and antibodies

Monoclonal antibodies (Mab) to hnRNP A1 (clone 9H10, Sigma-Aldrich, St. Louis, MO) or an isotype control IgG2b (Millipore MABF1079Z) buffer exchanged with PBS were conjugated with 10 mM XenoLight CF 680 dye with a degree of labeling (DOL) of approximately 4 (Perkin Elmer, Waltham, MA). 20 μg of these conjugates (‘A1_Mab_680’ or ‘control_IgG_680’) were injected into each mouse. 100 μL of a fluorescent imaging agent MMP750 Fast (‘MMP_750’) (Perkin Elmer, Waltham, MA), a matrix metalloproteinase activatable agent that recognizes MMPs 2, 3, 7, 9, 12 and 13 was used to examine MMP activity *ex* vivo [18].

### Induction and clinical evaluation of EAE

All animal procedures were reviewed and approved by the University of Tennessee and Veterans Affairs Medical Center–Memphis Institutional Animal Care and Use Committees (Protocol Number: 962165). Mice were housed on a 12:12 hour light:dark cycle, with standard chow and water freely available under pathogen-free conditions. A sentinel mouse was present in each room, which was routinely assessed for mouse-borne illnesses. A Th17 adoptive transfer model of EAE (‘Th17 EAE’) was utilized [9]. Briefly, C57BL/6 female mice (10–12 weeks old, Taconic farms, Rensselaer, NY) were immunized with MOG_35-55_(Hooke Laboratories, Lawrence, MA). Spleens from these actively immunized female C57BL/6 mice were extracted 11 days after MOG_35–55_ immunization, dissected and passed through a 70 μm filter (FalconTM) to obtain a single cell suspension. Following red blood cell lysis and centrifugation, 5 X 10^6^ cells/mL were cultured in the presence of MOG_35–55_ (20 μg/ml), TGF-β1(2 ng/ml), IL-6 (25 ng/ml), IL-23 (20 ng/ml), IL-1β (20 ng/ml), anti-IL-4 (10 μg/ml) and anti-IFN-γ (10 μg/ml). After 72 hours, Ficoll-gradient isolated splenocytes (20 x 10^6^)/mouse were injected intraperitoneally into recipient female C57BL/6 mice to induce adoptive transfer EAE. Samples of splenocytes used in the adoptive transfer were tested and confirmed to produce IL-17A by a commercial cytokine assay system (R and D systems, United Kingdom) (not shown). Four groups of mice (n = 5 each) were examined: (1) non-EAE (untouched control), (2) 5 days post-adoptive transfer (‘pre-disease’), (3) 13 days post-adoptive transfer (‘peak disease’) and (4) 21–28 days post-adoptive transfer (‘chronic disease’). Animals were examined daily and scored on a standardized five-point scale (1-limp tail, 2-partial hind limb paralysis, 3-complete hind limb paralysis, 4-complete hind limb & partial front limb paralysis, 5-moribund) [6, 9]. IACUC guidelines published by the University of Pennsylvania for EAE were used to outline the standard of care. Animals with clinical scores 1–3 were monitored for further deterioration of their condition and to ensure that there was adequate intake of food and water. The following alternate endpoints were used (a) severe dysfunction: clinical scores of 4 for three consecutive days were immediately euthanized, (b) greater than 20% loss of body weight over the course of the experiment were euthanized prior to the end of experimental procedure. However, no animals died prior to being euthanized at the appropriate experimental time point. For planned experimental end points, mice were euthanized using isoflurane overdose.

### *Ex vivo* fluorescent imaging analyses, quantification and statistical analyses

Injections of fluorescently labeled agents (A1_Mab_680, control_IgG_680 and MMP_750) were done retro-orbitally. After 24 hours, mice were sacrificed and the brain and spinal cord were dissected and transferred to a −80°C freezer. The mouse brain and spinal cord were then scanned using an *in vivo* imaging system (IVIS Lumina XR System, Perkin Elmer, Waltham, MA) with a high range filter set. The excitation and emission wavelengths used for MMP_750 were 745 nm and 800 nm, respectively, and for A1_Mab_680 and control_IgG_680 were 675 nm and 720 nm, respectively. To analyze fluorescence signal intensity, each brain and spinal cord were quantified using Living Image 4.0 software to calculate the average radiant efficiency omnidirectionally from the region of interest (ROI) [18]. Background fluorescence was removed by subtracting the fluorescence of null or background captured area. To analyze fluorescence signal location, individual images were imported into ImageJ. The entire length of the spinal cord (not including the cauda equina) was measured using the line tool. The length of each ROI was measured and then a measurement was taken from the bottom of the spinal cord to the middle of the ROI (S1A Fig) These measurements were imported into Microsoft Excel. In order to correct for differences in cord length between animals, the measurement from the middle of the ROI to the end of the cord was divided by total cord length, which resulted in a numerical position designation between 0.0 (bottom of cord) and 1.0 (top of cord) (S1B Fig). SPSS was used to run statistical analyses. One-way ANOVAs were used to test for (1) differences in clinical score across time, (2) differences in ROI average radiant efficiencies across time for each probe in the brain and spinal cords and (3) ROI displacement (or shift) relative to the bottom of the spinal cord, with Bonferroni post-hoc tests to determine differences between groups. P-values less than 0.05 were considered significant. A Pearson’s correlation analysis was used to determine whether there was a relationship between the MMP_750, A1_Mab_680, or control_IgG_680 markers with EAE clinical score in either the spinal cord. R values greater than 0.70 are considered strong, 0.50 considered moderate and p-values calculated for the correlations were considered significant if less than 0.05.

## Results

### *In vitro* fluorescence imaging using IVIS

**Fig 1:**
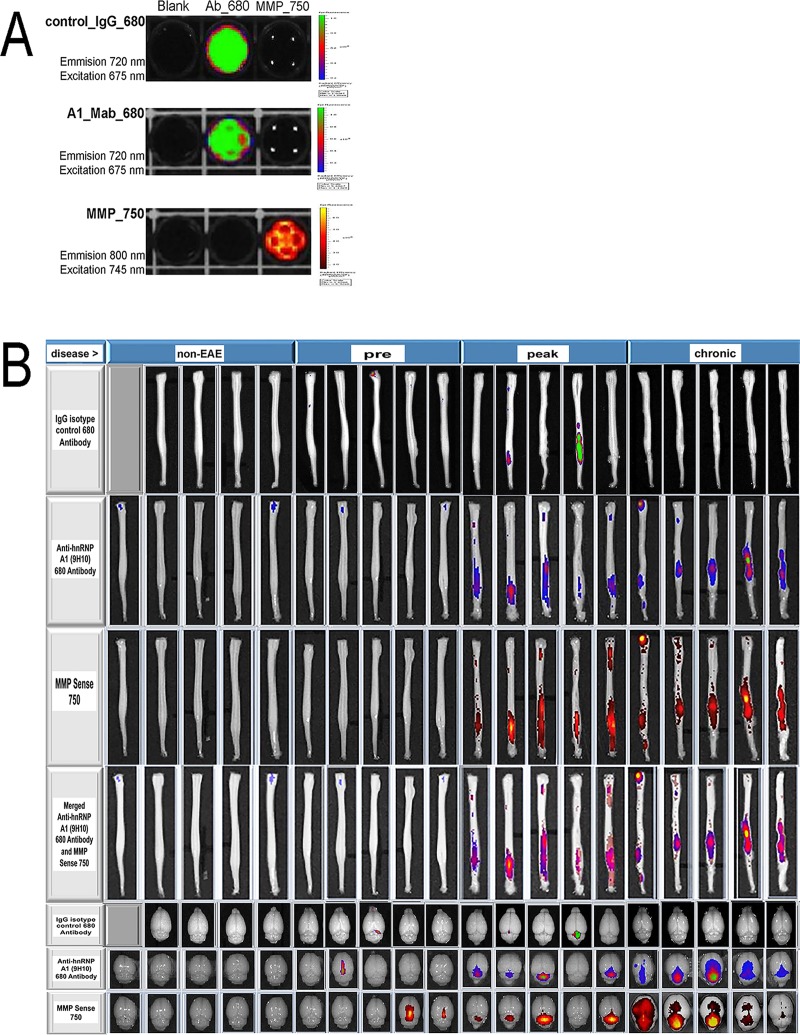
*In vitro* and *ex vivo* detection of control_IgG_680, A1_Mab_680 and MMP_750 fluorescence by IVIS imaging in EAE. (A) *In vitro* assessment of control IgG_680 shows a signal at the appropriate wavelength (row 1). In experiments that used both MMP_750 and A1_Mab_680 fluorescence in the same animal, there was no overlap in signal (rows 2 and 3). (B) *In vivo* imaging: four groups of mice (non-EAE, pre-disease, peak disease and chronic disease) were injected with either control_IgG_680 (rows 1 and 5) or A1_Mab_680 and MMP_750 (rows 2–4, 6 and 7) 24 hours prior to sacrifice. Clinical scores were obtained on the day of sacrifice. Row 1: Spinal cord control_IgG_680 imaging shows signal in two of five animals at the peak disease stage and marginal signal at other time points. Row 2: Spinal cord A1_Mab_680 imaging shows signal predominantly at the peak and chronic stages of EAE. Row 3: Spinal cord MMP_750 imaging closely parallels the A1_Mab_680 imaging. Row 4: Merged images of rows 3 and 4 show overlap between A1_Mab_680 and MMP_750 and a caudal to rostral shift of both signals. Row 5: Brain control_IgG_680 imaging shows modest signal in the same animals as the spinal cord imaging. Rows 6 and 7: Brain A1_Mab_680 and MMP_750 imaging (respectively) show increased signal in brain.

To confirm labelling of the antibodies and MMP_750, in vitro samples were examined at the appropriate wavelengths (Fig 1A). At an excitation wavelength of 675 nm and emission wavelength of 720 nm, both the control_IgG_680 and A1_Mab_680 were detected. In experiments that utilized both MMP_750 and A1_Mab_680 in the same animals, the two dyes were measured by IVIS scan simultaneously with different filter sets to assure the fluorescent signals did not interfere with each other. At an excitation wavelength of 745 nm and an emission wavelength of 800, MMP_750 (Fig 1A) was detected, whilst A1_Mab_680 was not. Similarly, at an excitation wavelength of 675 nm and an emission wavelength of 720 nm, the A1_Mab_680 was detected (Fig 1A) and the MMP_750 was not. Thus, each dye was detected at its respective wavelength without an overlapping signal.

### Clinical evaluation of EAE over time

**Fig 2:**
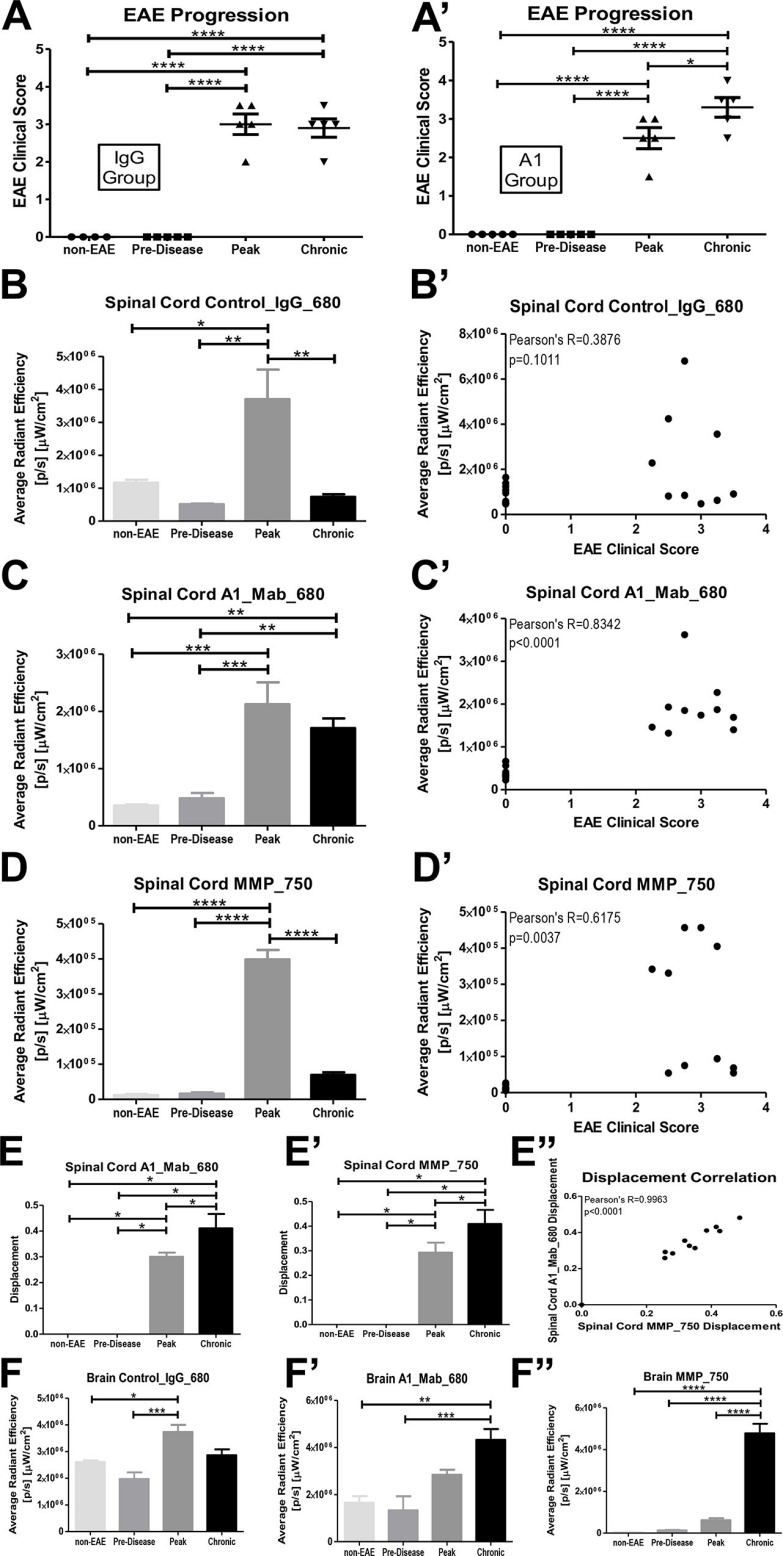
Quantitative analyses of *ex vivo* imaging in EAE. (A/A’): Both control_IgG _680 (A) and A1_Mab_680 (A’) injected animals developed clinical disease. (B/B’): In control_IgG_680 injected mice, there was significantly increased average radiant efficiency in spinal cord during peak disease compared to non-EAE, pre-disease and chronic disease (B) and no correlation between average radiant efficiency and EAE clinical score (B’). (C/C’): In A1_Mab_680 injected mice, there was significantly increased average radiant efficiency in spinal cord during peak and chronic disease compared to non-EAE and pre-disease (C) and a strong correlation between average radiant efficiency and EAE clinical score (C’). (D/D’): In MMP_750 injected mice, there was significantly increased average radiant efficiency in spinal cord during peak disease compared to non-EAE, pre-disease and chronic disease (D) and modest correlation between average radiant efficiency and EAE clinical score (D’). (E/E’/E”): In spinal cord, both A1_Mab_680 and MMP_750 fluorescence showed a rostral to caudal displacement (E/E’) and a strong correlation between the two measurements (E”). (F/F’/F”): In brain, there was increased average radiant efficiency in the IgG_Mab_680 injected mice at peak disease (F), increased average radiant efficiency of A1_ Mab_680 in the chronic stage of disease (F’) and increased average radiant efficiency of MMP_750 in the chronic phase of disease (F”). (*p<0.05, **p<0.01, ***p<0.001, ****p<0.0001).

Th17 EAE mice from both the control_IgG_680 and A1_Mab_680 injected groups developed EAE (Fig 2A and 2A’). There was a statistically significant worsening of clinical scores when comparing the peak disease and chronic disease with pre-disease and non-EAE groups, consistent with other studies using this model [9].

### *Ex vivo* fluorescence imaging of spinal cord and brain in EAE using IVIS

Following injection of control_IgG_680 antibodies (Fig 1B, row 1, top), there was marginal fluorescent signal in spinal cord in either the non-EAE or pre-disease groups. Two of five mice showed signal at peak disease and no mice showed signal in the chronic phase of EAE (Fig 1B, row 1, top). Quantification of fluorescence over time showed statistically significant differences in average radiant efficiency when comparing peak disease with both the pre-disease and non-EAE groups (Fig 2B). There was no difference in average radiant efficiency between the chronic and pre-disease or non-EAE groups. Importantly, there was a significant reduction in average radiant efficiency when comparing chronic with peak disease, indicating that the control_IgG_680 antibody did not persist in the spinal cord between the peak and chronic stages of Th17 induced EAE (Fig 2B). Furthermore, there was no correlation between EAE clinical score and average radiant efficiency (Fig 2B’).

We next examined the injection of the A1_Mab_680 antibodies and MMP_750, a marker of neuroinflammation. Following injection of both A1_Mab_680 and MMP_750, there was marginal fluorescent signal in the non-EAE and pre-disease groups (Fig 1B–rows 2 and 3). In contrast, as the animals became ill over time, there was an increase in fluorescent signal of both markers in the spinal cords of the animals (Fig 1B–rows 2 and 3).

Quantification of fluorescence over time in the spinal cords, showed a statistically significant increase in A1_Mab_680 average radiant efficiency when comparing either the peak disease or chronic disease with the pre-disease and non-EAE groups (Fig 2C). There was no difference in average radiant efficiency between the peak and chronic disease groups, indicative of the A1_Mab_680 antibody’s persistence from peak to chronic disease. There was a strong correlation between EAE clinical score and average radiant efficiency in the mice injected with A1_Mab_680 antibodies (Fig 2C’). MMP_750 fluorescent signal was maximal at peak disease compared to non-EAE, pre-disease and chronic disease (Fig 1B–row 3 and Fig 2D). There was a modest correlation between MMP_750 average radiant efficiency and EAE clinical score (Fig 2D’).

Next, we observed that between peak and chronic disease, there was a caudal to rostral shift of both the A1_Mab_680 and MMP_750 fluorescent signals in the distal spinal cord (Fig 1B–rows 2 and 3). Statistical analyses of the fluorescent ROIs showed a significant caudal to rostral displacement (shift) of both signals (Fig 2E and 2E’). There was a statistically strong correlation (overlap of location) between MMP_750 and A1_Mab_680 ROIs (Fig 1B–row 4 and Fig 2E”).

In the control_IgG_680 injected group, the same mice that showed fluorescent signal in spinal cord at peak disease also showed signal in brain (Fig 1B–row 5, Fig 2F). In brain, fluorescence of both A1_Mab_680 and MMP_750 increased significantly over time (Fig 1B–rows 6 and 7, Fig 2F’ and 2F”). Interestingly, signal was highest in the cerebellar/brainstem area (Fig 1B–rows 6 and 7).

## Discussion

In this study, we were able to reliably and reproducibly quantify the fluorescent signal of near-IR labeled antibodies, which localized to the CNS of mice with EAE. There was a strong correlation between anti-hnRNP A1 antibody signal and disease activity and the antibody signal persisted between the peak and chronic phase of EAE. In contrast, there was no correlation between the isotype control IgG signal and disease activity and the control antibodies did not persist between the peak and chronic phase of disease. Taken together, these data suggest that the anti-hnRNP A1 antibodies specifically bound a CNS target in mice, while the control antibodies entered the CNS non-specifically.

We also showed that MMP_750 and A1_Mab_680 fluorescent signals could be detected without interference from each other, thus assuring the uniqueness of each signal when injected simultaneously. Using this technique, we showed that A1_Mab_680 antibodies localized to the CNS and both MMP_750 and A1_Mab_680 fluorescent signals increased over time. As the animals became sicker, both signals increased and correlated with EAE clinical score. The A1_Mab_680 correlated more strongly, suggesting it may be more sensitive in detecting disease severity in EAE.

In addition, we also showed that there was a caudal to rostral shift of both signals over time, consistent with EAE being an ascending paralysis [6]. Finally, not only could these signals be visualized in spinal cord, we also showed that they localized to brain.

MMPs have been shown to contribute to the pathogenesis of neuroinflammatory diseases including MS and EAE [11, 12, 19–21]. MMP_750 detects the activities of MMP 2, 3, 7, 9, 12 and 13. MMPs 2 and 9 have been shown to be particularly relevant to MS and EAE [19]. In MS, CSF levels of MMP-9 are increased in relapsing forms of MS and CSF and serum levels of MMP-9 inversely correlate with clinical improvement following treatment with beta-interferon 1ß [19]. MMP-2 and MMP-9 have been implicated in the pathogenesis of EAE including their role in neuroinflammation, leukocyte migration into the CNS and blood brain barrier (BBB) disruption [11, 12, 19, 21]. It is this latter function that we think is particularly relevant to this study. Considering that the A1_Mab_680 is an IgG, BBB disruption is important for its entry into the CNS, thus the strong correlation between the A1_Mab_680 and MMP_750 signals. Interestingly, the A1_Mab_680 signal in the spinal cord was maintained beyond that of MMP_750, suggesting it persists in the CNS following acute neuroinflammation.

Dysfunctional ribonucleoproteins, including hnRNP A1, have been shown to cause a number of neurological diseases including frontotemporal lobe dementia and amyotrophic lateral sclerosis [22]. The role of hnRNP A1 and similar ribonucleoproteins in MS is just beginning to be understood [1, 6, 7]. Interestingly, our laboratory has shown that MS patients develop antibodies to hnRNP A1, and specifically to its M9 sequence [8]. M9 is a crucial region of hnRNP A1, as it is required to shuttle hnRNP A1 into and out of the nucleus. Using EAE, our lab has shown that anti-hnRNP A1-M9 Mabs (the same antibodies used in this study) worsened disease, caused spasticity and increased levels of CNS neurodegeneration [7]. Specifically, we found that anti-hnRNP A1-M9 Mabs caused increased levels of neurodegeneration in the distal projection of the ventral spinocerebellar tract (VSCT) [7]. The VSCT cells of origin are in the lumbosacral spinal cord of the mouse and its axons project via the brainstem to the cerebellum [7]. Remarkably, these are the same areas where the A1_Mab_680 preferentially localized in this study. Although an exact mechanism of how the anti-hnRNP A1-M9 antibodies cause neurodegeneration is under study and incompletely understood, the fact that their macroscopic localization in EAE correlates with both the cells of origin and distal synapse of the tract, is an important observation. This is relevant to MS, as antibodies have been shown to contribute to the pathogenesis of MS and both anti-B-cell therapies and plasma exchange have shown efficacy in treating MS [17, 23].

## Conclusion

In summary, we successfully localized near-IR fluorescently-labeled antibodies to the CNS of EAE. The anti-hnRNP A1 signal localized to the CNS, colocalized with MMP activity, correlated with clinical disease and shifted rostrally within the spinal cord consistent with EAE being an ascending paralysis. Considering the contribution of antibodies to the pathogenesis of MS, this technique can be used to detect, localize and assess the pathogenesis of other antibodies that may play a role in autoimmune diseases of the CNS.

## Supporting information

S1 Fig **A**. To analyze fluorescent signal location, individual images were imported into ImageJ. The entire length of the spinal cord was measured using the line tool. Then the ROI was measured in length and a measurement was taken from the bottom of the spinal cord to the middle of the ROI. These measurements were imported into Microsoft Excel. **B.** In order to correct for differences in cord length between animals, the measurement from the middle of the ROI to the end of the cord was divided by total cord length, which resulted in a numerical position designation between 0.0 (bottom of cord) and 1.0 (top of cord).
(DOCX)

## References

[1] Salapa H.E., Lee S., Shin Y., Levin M.C., Contribution of the Degeneration of the Neuro-Axonal Unit to the Pathogenesis of Multiple Sclerosis, Brain sciences 7 (2017) 69. 10.3390/brainsci7060069 PMC5483642 28629158

[2] Frischer J.M., Bramow S., Dal-Bianco A., Lucchinetti C.F., Rauschka H., Schmidbauer M., Laursen H., Sorensen P.S., Lassmann H., The relation between inflammation and neurodegeneration in multiple sclerosis brains, Brain 132 (2009) 1175–1189. 10.1093/brain/awp070 19339255 PMC2677799

[3] Antel J., Antel S., Caramanos Z., Arnold D.L., Kuhlmann T., Primary progressive multiple sclerosis: part of the MS disease spectrum or separate disease entity?, Acta Neuropathol 123 (2012) 627–638. 10.1007/s00401-012-0953-0 22327362

[4] Levin M.C., Lee S., Gardner L.A., Shin Y., Douglas J.N., Cooper C., Autoantibodies to Non-myelin Antigens as Contributors to the Pathogenesis of Multiple Sclerosis, J Clin Cell Immunol 4 (2013). 10.4172/2155-9899.1000148 PMC3866957 24363960

[5] Fraussen J., Claes N., de Bock L., Somers V., Targets of the humoral autoimmune response in multiple sclerosis, Autoimmun Rev 13 (2014) 1126–1137. 10.1016/j.autrev.2014.07.002 25108168

[6] Douglas J.N., Gardner L.A., Salapa H.E., Lalor S.J., Lee S., Segal B.M., Sawchenko P.E., Levin M.C., Antibodies to the RNA-binding protein hnRNP A1 contribute to neurodegeneration in a model of central nervous system autoimmune inflammatory disease, Journal of neuroinflammation 13 (2016) 178 10.1186/s12974-016-0647-y 27391474 PMC4938923

[7] Douglas J.N., Gardner L.A., Salapa H.E., Levin M.C., Antibodies to the RNA Binding Protein Heterogeneous Nuclear Ribonucleoprotein A1 Colocalize to Stress Granules Resulting in Altered RNA and Protein Levels in a Model of Neurodegeneration in Multiple Sclerosis, Journal of Clinical & Cellular Immunology 7 (2016) 402. 27375925 10.4172/2155-9899.1000402 PMC4928374

[8] Lee S., Xu L., Shin Y., Gardner L., Hartzes A., Dohan F.C., Raine C., Homayouni R., Levin M.C., A potential link between autoimmunity and neurodegeneration in immune-mediated neurological disease, J Neuroimmunol 235 (2011) 56–69. 10.1016/j.jneuroim.2011.02.007 21570130

[9] Carbajal K.S., Mironova Y., Ulrich-Lewis J.T., Kulkarni D., Grifka-Walk H.M., Huber A.K., Shrager P., Giger R.J., Segal B.M., Th Cell Diversity in Experimental Autoimmune Encephalomyelitis and Multiple Sclerosis, Journal of immunology (Baltimore, Md.: 1950) (2015). 10.4049/jimmunol.1501097 PMC4561206 26238492

[10] Berger C., Gremlich H.U., Schmidt P., Cannet C., Kneuer R., Hiestand P., Rausch M., Rudin M., In vivo monitoring the fate of Cy5.5-Tat labeled T lymphocytes by quantitative near-infrared fluorescence imaging during acute brain inflammation in a rat model of experimental autoimmune encephalomyelitis, J Immunol Methods 323 (2007) 65–77. 10.1016/j.jim.2007.02.009 17433359

[11] Eaton V.L., Vasquez K.O., Goings G.E., Hunter Z.N., Peterson J.D., Miller S.D., Optical tomographic imaging of near infrared imaging agents quantifies disease severity and immunomodulation of experimental autoimmune encephalomyelitis in vivo, J Neuroinflammation 10 (2013) 138 10.1186/1742-2094-10-138 24237884 PMC4225609

[12] Schmitz K., Tegeder I., Bioluminescence and Near-infrared Imaging of Optic Neuritis and Brain Inflammation in the EAE Model of Multiple Sclerosis in Mice, J Vis Exp (2017). 10.3791/55321 PMC5408958 28287595

[13] Sagar D., Lamontagne A., Foss C.A., Khan Z.K., Pomper M.G., Jain P., Dendritic cell CNS recruitment correlates with disease severity in EAE via CCL2 chemotaxis at the blood-brain barrier through paracellular transmigration and ERK activation, J Neuroinflammation 9 (2012) 245 10.1186/1742-2094-9-245 23102113 PMC3533869

[14] Ayzenberg I., Schlevogt S., Metzdorf J., Stahlke S., Pedreitturia X., Hunfeld A., Couillard-Despres S., Kleiter I., Analysis of neurogenesis during experimental autoimmune encephalomyelitis reveals pitfalls of bioluminescence imaging, PloS one 10 (2015) e0118550 10.1371/journal.pone.0118550 25780928 PMC4363373

[15] Wang C., Wu C., Popescu D.C., Zhu J., Macklin W.B., Miller R.H., Wang Y., Longitudinal near-infrared imaging of myelination, The Journal of neuroscience: the official journal of the Society for Neuroscience 31 (2011) 2382–2390. 21325505 10.1523/JNEUROSCI.2698-10.2011 PMC3044505

[16] Luo J., Ho P., Steinman L., Wyss-Coray T., Bioluminescence in vivo imaging of autoimmune encephalomyelitis predicts disease, J Neuroinflammation 5 (2008) 6 10.1186/1742-2094-5-6 18237444 PMC2267451

[17] Gelfand J.M., Cree B.A.C., Hauser S.L., Ocrelizumab and Other CD20(+) B-Cell-Depleting Therapies in Multiple Sclerosis, Neurotherapeutics: the journal of the American Society for Experimental NeuroTherapeutics 14 (2017) 835–841. 28695471 10.1007/s13311-017-0557-4 PMC5722762

[18] Cho H., Bhatti F.U., Lee S., Brand D.D., Yi A.K., Hasty K.A., In Vivo Dual Fluorescence Imaging to Detect Joint Destruction, Artif Organs 40 (2016) 1009–1013. 10.1111/aor.12685 27183538 PMC7060000

[19] Kandagaddala L.D., Kang M.J., Chung B.C., Patterson T.A., Kwon O.S., Expression and activation of matrix metalloproteinase-9 and NADPH oxidase in tissues and plasma of experimental autoimmune encephalomyelitis in mice, Exp Toxicol Pathol 64 (2012) 109–114. 10.1016/j.etp.2010.07.002 20810258

[20] Hannocks M.J., Zhang X., Gerwien H., Chashchina A., Burmeister M., Korpos E., Song J., Sorokin L., The gelatinases, MMP-2 and MMP-9, as fine tuners of neuroinflammatory processes, Matrix Biol (2017). 10.1016/j.matbio.2017.11.007 29158162

[21] Rosenberg G.A., Matrix metalloproteinases and neuroinflammation in multiple sclerosis, Neuroscientist 8 (2002) 586–595. 10.1177/1073858402238517 12467380

[22] Kim H.J., Kim N.C., Wang Y.D., Scarborough E.A., Moore J., Diaz Z., MacLea K.S., Freibaum B., Li S., Molliex A., Kanagaraj A.P., Carter R., Boylan K.B., Wojtas A.M., Rademakers R., Pinkus J.L., Greenberg S.A., Trojanowski J.Q., Traynor B.J., Smith B.N., Topp S., Gkazi A.S., Miller J., Shaw C.E., Kottlors M., Kirschner J., Pestronk A., Li Y.R., Ford A.F., Gitler A.D., Benatar M., King O.D., Kimonis V.E., Ross E.D., Weihl C.C., Shorter J., Taylor J.P., Mutations in prion-like domains in hnRNPA2B1 and hnRNPA1 cause multisystem proteinopathy and ALS, Nature 495 (2013) 467–473. 10.1038/nature11922 23455423 PMC3756911

[23] Keegan M., Konig F., McClelland R., Bruck W., Morales Y., Bitsch A., Panitch H., Lassmann H., Weinshenker B., Rodriguez M., Parisi J., Lucchinetti C.F., Relation between humoral pathological changes in multiple sclerosis and response to therapeutic plasma exchange, Lancet 366 (2005) 579–582. 10.1016/S0140-6736(05)67102-4 16099294

