# Supplementary material for: Localization of near-infrared labeled antibodies to the central nervous system in experimental autoimmune encephalomyelitis
Source: PLoS One. 2019 Feb 15;14(2):e0212357. doi: 10.1371/journal.pone.0212357 (PMC6377130; doi:10.1371/journal.pone.0212357)
Supplement: S1 Fig — A. To analyze fluorescent signal location, individual images were imported into ImageJ. The entire length of the spinal cord was measured using the line tool. Then the ROI was measured in length and a measurement was taken from the bottom of the spinal cord to the middle of the ROI. These measurements were imported into Microsoft Excel. B. In order to correct for differences in cord length between animals, the measurement from the middle of the ROI to the end of the cord was divided by total cord length, which resulted in a numerical position designation between 0.0 (bottom of cord) and 1.0 (top of cord). (DOCX) [file pone.0212357.s001.docx]

**Supplement Figure 1A**. To analyze fluorescent signal location, individual images were imported into ImageJ. The entire length of the spinal cord was measured using the line tool. Then the ROI was measured in length and a measurement was taken from the bottom of the spinal cord to the middle of the ROI. These measurements were imported into Microsoft Excel.


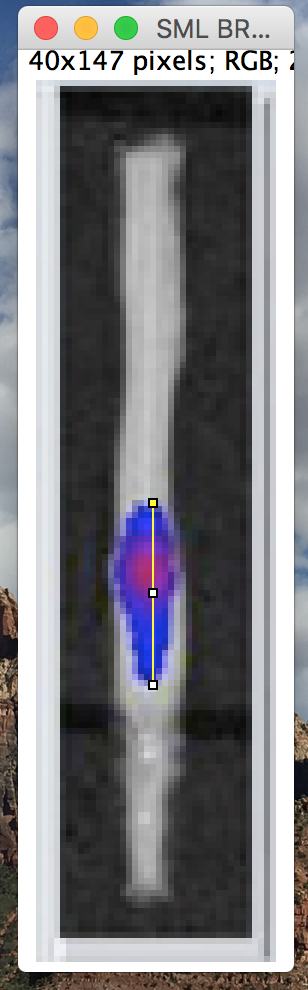

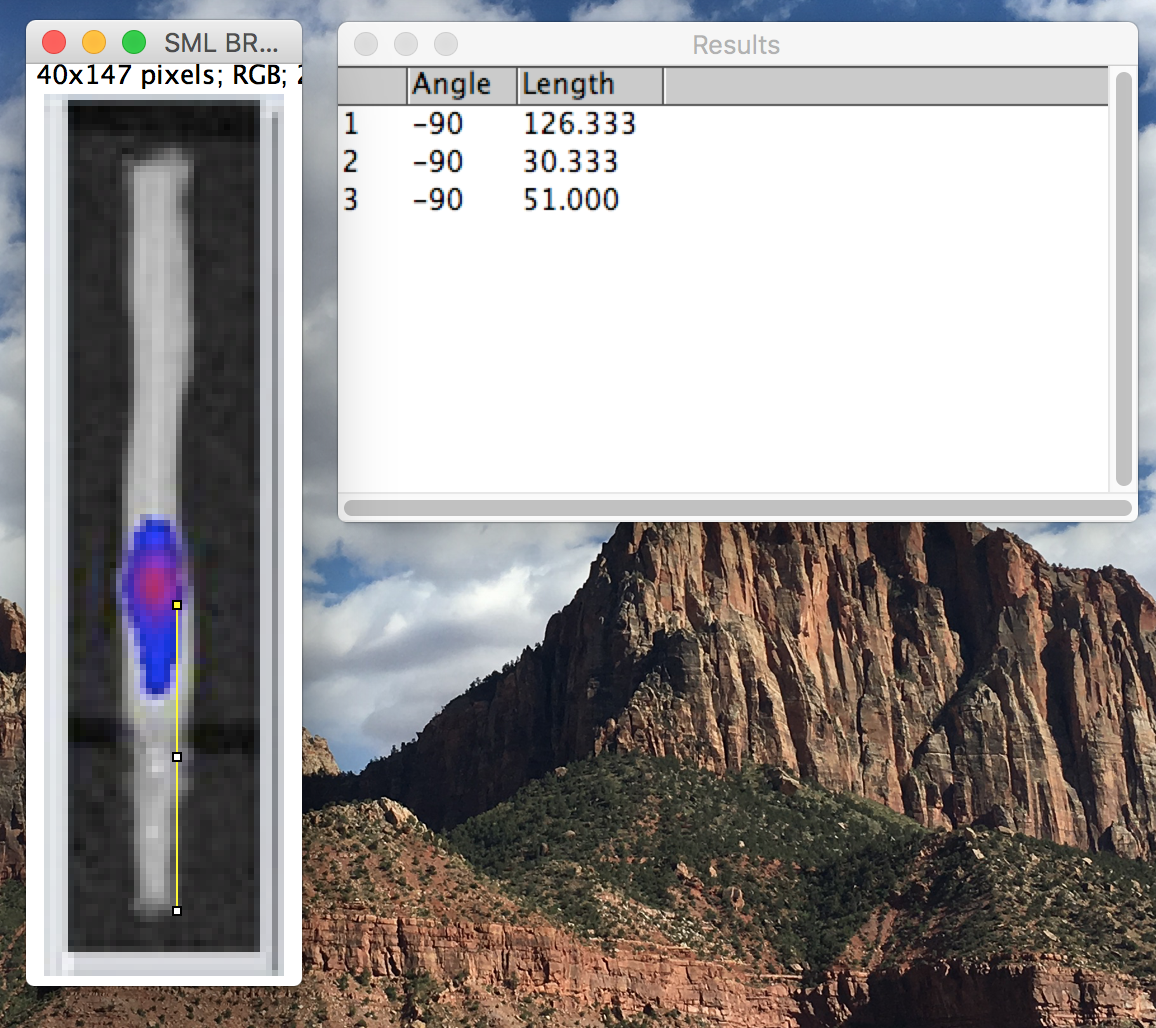

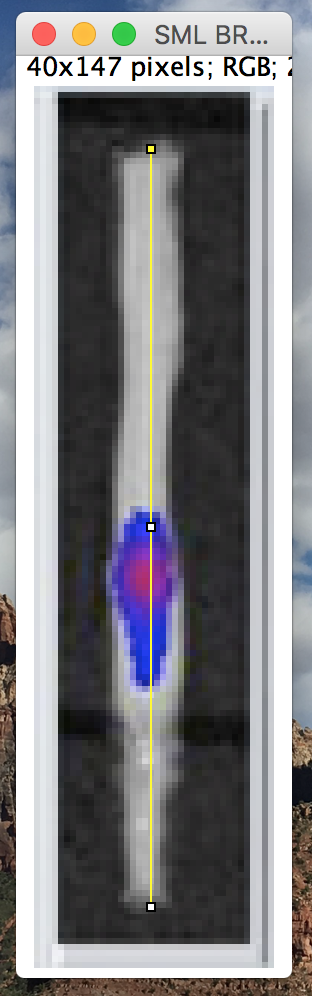


**Supplement Figure 1B.** In order to correct for differences in cord length between animals, the measurement from the middle of the ROI to the end of the cord was divided by total cord length, which resulted in a numerical position designation between 0.0 (bottom of cord) and 1.0 (top of cord).


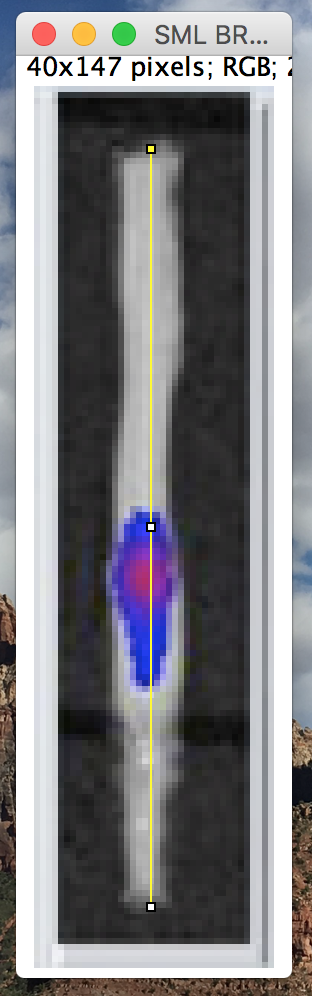


🡨 1.000

🡨 0.000
